# Status and global population trend of the Magellanic penguin *Spheniscus magellanicus* along the Argentine coast

**DOI:** 10.1038/s41598-025-33756-3

**Published:** 2026-01-10

**Authors:** Jesica D. Hombre, Magdalena Arias, Mauro F. Carrasco, Raúl A. C. González, Enrique A. Crespo

**Affiliations:** 1 Centro de Investigación Aplicada y Transferencia Tecnológica en Recursos Marinos Almirante Storni (CIMAS, CONICET), San Antonio Oeste, Río Negro Argentina; 2 Facultad de Ciencias Marinas (FACIMAR, Universidad Nacional del Comahue), San Antonio Oeste, Río Negro Argentina; 3 Universidad Tecnológica Nacional. Facultad Regional Chubut. Grupo de Investigación en Energía, Materiales y Sustentabilidad, San Antonio Oeste, Río Negro Argentina; 4 Centro de Estudios de Sistemas Marinos (CESIMAR, CENPAT, CONICET), Puerto Madryn, Chubut Argentina; 5 Centro de Ciencias Naturales, Ambientales y Antropológicas, Universidad Maimónides y Fundación Azara, Ciudad Autónoma de Buenos Aires, Argentina

**Keywords:** *Spheniscus magellanicus*, Magellanic penguin, Population trends, Breeding colonies, Long-term monitoring, Southwest Atlantic, Ecology, Ecology, Ocean sciences

## Abstract

The Magellanic penguin *Spheniscus magellanicus* is one of the emblematic species of the Argentine Atlantic coast, and its population dynamics provide valuable information for coastal ecosystem monitoring. Despite its wide distribution, colony abundance and spatial configuration along the Argentine coast have undergone significant changes in recent decades. This study aims to characterize the current status and trends of breeding colonies throughout its distribution along the Argentine Atlantic coast. Together with our own surveys we conducted an exhaustive literature review, integrating more than three decades of published and unpublished information on 65 active breeding colonies distributed from Río Negro to Tierra del Fuego, including the Malvinas/Falkland Islands. Abundance estimates were compiled and analyzed by colony, allowing for the evaluation of individual trends and the estimation of an overall population trend for the species in the country. While trends varied among individual colonies, the overall results indicate a moderate, although not statistically significant, multi-decadal population increase of approximately 2% per year, based on colony-specific trends derived from long-term monitoring data. Based on individual colony trends, total abundances were estimated, projecting approximately 1.28 million breeding pairs by the year 2024. Although regional differences were found, there is a sustained increase in the total number of active colonies, an expansion into new areas, and growth in several already established colonies. This result contrasts with previous perceptions of widespread decline and reinforces evidence of a reconfiguration in the species’ breeding distribution. These changes respond to a combination of ecological factors, dispersal processes, and variations in anthropogenic pressure. The findings highlight the importance of maintaining systematic monitoring programs and adapting conservation strategies to the spatial and temporal dynamism observed.

**Supplementary Information:**

The online version contains supplementary material available at 10.1038/s41598-025-33756-3.

## Introduction

The Magellanic penguin *Spheniscus magellanicus* is a seabird species endemic to the Southern Cone of South America, nesting in colonies along the coasts of Argentina, Chile, and the Malvinas/Falkland Islands. This long-lived, colonial and highly philopatric species breeds annually between September and February, nesting in caves, under bushes or in natural cavities in coastal environments^1–3^. During the austral winter, a large part of the population migrates north, reaching as far south as Uruguay and southern Brazil^4,5^.

Currently, its distribution in Argentina extends from Islote Lobos National Park (Río Negro) to Isla Martillo in the Beagle Channel (Tierra del Fuego), also including island colonies on Isla de los Estados and the Malvinas/Falkland Islands^6,7^. More than 60% of the world’s breeding population of this species is found in Argentina, which confers a particular conservation responsibility^8,1,9,5^.

In the southwestern Atlantic, interactions between Magellanic penguins and commercial fisheries have been highlighted as a potential conservation concern. These interactions can be grouped into two main categories: (1) operational, involving direct mortality through incidental capture in fishing gear, particularly trawls and gillnets, and (2) ecological, arising from trophic overlap when fisheries target species that are key prey for penguins^10–13^. The species’ diet varies according to the latitudinal location of colonies: Scolaro et al.^14^ reported that penguins in northern colonies (Chubut) consumed primarily anchovy (*Engraulis anchoita*), while individuals in southern colonies (Santa Cruz) fed mainly on squid (*Loligo* spp. and *Illex* spp.), Fuegian sprat (*Sprattus fuegensis*), and hagfish (*Myxine* spp.). Fernández et al.^15^ found that penguins in colonies located further north (San Matías Gulf) fed mainly on anchovy, tornfish (*Bovichtus argentinus*), Patagonian rockfish (*Sebastes oculatus*), and squat lobster (*Munida gregaria*). Gandini et al.^16^, based on penguins caught as bycatch in the Patagonian red shrimp fishery, also reported the presence of Argentine hake (*Merluccius hubbsi*), small octopus (*Octopus* sp.), prawn (*Peisos petrunkevitchi*), mantis shrimp (*Stomatopoda*), and crabs (Brachyura), in addition to anchovies, squids, and squat lobsters. Several of these species are target resources for regional fisheries, which has been proposed as a potential source of food competition. In scenarios of low prey availability, such competition could affect foraging success, reproduction, and ultimately, penguin population dynamics^16,17^.

The oldest documented references to the species in Argentine territory come from the records of 16th-century European explorers, such as Francis Drake, Thomas Cavendish, and Olivier Van Noort^18^. These accounts described large groups of flightless seabirds used as a food source in the Strait of Magellan and surrounding areas^18^. However, recent continental archaeological evidence indicates that the breeding presence of this species in continental Argentina is a relatively recent phenomenon: excavations at sites such as the Cabo Vírgenes Provincial Reserve have not found skeletal remains from before the 20th century^18^. Radiocarbon dating of a penguin tarsometatarsus recovered from the site yielded an age of only 105 years, suggesting that its continental presence is a recent phenomenon in ecological terms^18^. It has been proposed that the species originally nested primarily on islands, where the absence of terrestrial predators offered safer breeding conditions^4^. The expansion of livestock farming, the transformation of the coastal landscape, and the elimination of native predators such as foxes and pumas may have facilitated the colonization of previously unsuitable continental areas, a process that accelerated during the 20th century^18,7^. Modern scientific knowledge of *S. magellanicus* in Argentina began to consolidate in the second half of the 20th century. In the 1950s, Carrara^19^ conducted the first systematic surveys along the coast from Buenos Aires to Tierra del Fuego. Later, Scolaro and Kovacs^20^ documented the appearance of continental colonies in northern Chubut and the Valdés Peninsula. During the 1980s and 1990s, one of the most extensive ecological monitoring programs, including an internationally benchmark reproductive and population parameters for this species at Punta Tombo was established by Boersma et al.^21^. On the other hand, significant colony growth was documented in the 1990s at Estancia San Lorenzo and Caleta Externa, both located in the Valdés Peninsula^22^. In Santa Cruz province, long-term monitoring programs have been conducted in key areas such as Puerto Deseado^9,23^. Meanwhile, in Tierra del Fuego province, new colonies have been reported on sub-Antarctic islands, such as San Juan de Salvamento, suggesting active colonization of previously unoccupied remote areas^24^. In parallel, the species has shown a progressive expansion towards the north of the Argentine coast. The growth and establishment of colonies in northern Chubut has been interpreted as part of an active metapopulation dynamic in the region^25^. The colony in Islote Lobos National Park (41°26’S, 65°00’W), is currently the northernmost known. Its reproductive presence was first recorded in 2002 during field surveys conducted by García Borboroglu et al.^26^, who documented the nesting of 22 active pairs. Although it has not been systematically monitored since its discovery, since 2022 our team has begun annual surveys with the aim of understanding its population and ecological dynamics. The establishment of this colony marked a new step in the northward expansion of the species’ breeding distribution, extending its range to the province of Río Negro for the first time. This record aligns with the previously documented expansion in northern Chubut^7,25^ and supports a broader trend of progressive colonization toward more northerly sectors of the Argentine coastline. In this regard, it is also relevant to consider the breeding colonies present in the Malvinas/Falkland Islands, an Argentine island territory in the South Atlantic. Although there is controversy among sources, according to^27,28^, these colonies have experienced a marked population decline in recent decades, primarily attributed to intensive fishing activity in the region since the late 1980s. Despite their ecological importance within the marine-coastal system of the southwest Atlantic, scientific coverage of these colonies has focused on foreign research. Including this region in a national analysis of abundance and population trends allows for a more comprehensive view of the species’ conservation status throughout its entire distribution range in Argentina. In this context, this study aims to estimate the total abundance and population trends of the Magellanic penguin along the entire Argentine coast, integrating recent field data and historical records, and to provide a clearer and more up-to-date view of its population status and changes over recent decades. Despite the volume of regional studies conducted in recent decades, a significant gap persists in the comprehensive analysis of the species’ current status in Argentina. Most available assessments have focused on specific colonies or provinces, often with limited temporal coverage^1,25,29^. It is known that some colonies are increasing in numbers, some are declining and some remain stable^1,25,29^. Therefore, the main goal of this paper consists of carrying a global analysis of the population in order to make clear the population trend and abundance of the species in Argentine waters. This goal is important given that the species has been considered to be subject to anthropomorphic risks like interaction with fisheries, oil pollution, climate change and habitat degradation.

## Materials and methods

### Literature review

Information on the abundance of Magellanic penguins along different regions of the Argentine coast was compiled from scientific literature, technical reports, theses, monitoring documents from governmental and non-governmental organizations, and our own field data. The literature search was conducted primarily through Google Scholar using combinations of the terms “Magellanic penguin”, “Spheniscus magellanicus”, “colony”, “abundance”, “population size”, and the names of provinces or breeding sites. Additional documents were identified by screening the reference lists of key publications.

Only records reporting quantitative estimates of abundance expressed as the number of breeding pairs were retained, ensuring consistency and full comparability across datasets. For each document, we extracted the colony name, geographic location, survey year(s), abundance estimate, and a short description of the census methodology when available.

The abundance database was compiled from published studies, technical reports, and long-term monitoring programs listed in Table 1 (e.g. ^1,7–9,14,20,22,24–26,28–39,58^).

**Table 1:** Total number of colonies analyzed.

	**Colony**	**ID**	**Location**	**Province**	**r**	**λ**
**1**	Bahía San Antonio^a,b^	**BSA**	40°47’S, 64°47’W	Río Negro	-0.0101	0.99 (0.74 – 1.35)
**2**	Islote La Pastosaa,d,e	**ILP**	41°25’S, 65°02’W	Río Negro	0.0159	1.02 (0.96 – 1.08)
**3**	Islote Redondoc,d,e	**IR**	41°26’S, 65°01’W	Río Negro	0.1970	1.22(1.13 – 1.31)*
**4**	Isla de los Pájarose	**IP**	41°27’S, 65°02’W	Río Negro	-0.0896	0.91 (0.79 – 1.06)
**5**	Punta Pozos^a^	**PP**	41°34’S, 65°01’W	Río Negro	-0.3164	0.73
**6**	Islote Notable^c,f,g,h,i^	**IN**	42°25′S, 64°31′W	Chubut	0.1768	1.19 (1.10 – 1.29)*
**7**	Estancia San Lorenzo^a,c,d,g,h,j,k,l^	**ESL**	42°05’S, 63°51’W	Chubut	0.1989	1.22 (1.16 – 1.28)*
**8**	Asentamiento Oeste^a,c,g,h,i^	**AO**	42°06’S, 63°56’W	Chubut	0.0441	1.05 (1.01 – 1.09)*
**9**	Caleta Externa^a,c,d,g,h,i^	**CE**	42°16’S, 63°38’W	Chubut	0.0916	1.10 (1.07 – 1.13)*
**10**	Isla Primera^a,c,g,i^	**I1°**	42°21′S, 63°37′W	Chubut	0.0451	1.05 (1.02 – 1.07)*
**11**	Isla Segunda^a,c,g,h,i^	**I2°**	42°20′S, 63°39′W	Chubut	0.1474	1.16 (1.16 – 1.20)*
**12**	Caleta Interna^a,c,g,h,i,m,n^	**CI**	42°27′S, 63°36′W	Chubut	-0.0179	0.98 (0.96 – 1.01)
**13**	El Pedral^a,d,p^	**EP**	42°57′S, 64°23′W	Chubut	0.5622	1.76 (1.42 – 2.11)*
**14**	Punta Clara^a,h^	**PC**	43°58′S, 65°15′W	Chubut	-0.0188	0.98
**15**	Punta Tombo^a,d,h,m,p,q^	**PT**	44°02′S, 65°11′W	Chubut	-0.0167	0.98 (0.98 – 0.99)*
**16**	Punta Lobería^a,h^	**PL**	44°35′S, 65°22′W	Chubut	0.0445	1.05
**17**	Isla Blanca Mayor^a,h^	**IBM**	44°46′S, 65°38′W	Chubut	-0.0403	0.96
**18**	Cabo Dos Bahías^a,d,r^	**CDB**	44°54′S, 65°32′W	Chubut	-0.0030	1.00 (0.99 – 1.01)
**19**	Isla Arce^a,r^	**IA**	45°00′S, 65°29′W	Chubut	-0.0820	0.92
**20**	Isla Leones^a,d,r,s^	**IL**	45°03′S, 65°36′W	Chubut	-0.0675	0.94 (0.92 – 0.95)*
**21**	Península Lanaud^a,d,r,s^	**Pla**	45°03′S, 65°35′W	Chubut	-0.0044	1.00
**22**	Isla Buque^a,r,s^	**IB**	45°03′S, 65°37′W	Chubut	-0.0236	0.98
**23**	Isla Sudoeste^a,r,s^	**ISO**	45°03′S, 65°36′W	Chubut	-0.0778	0.93
**24**	Isla Tova^a,r^	**IT**	45°06′S, 66°00′W	Chubut	-0.0231	0.98
**25**	Isla Tovita^a,r,s^	**ITa**	45°07′S, 65°57′W	Chubut	-0.0631	0.94 (0.92 – 0.96)*
**26**	Isla Este^a,c,r^	**IE**	45°07′S, 65°56′W	Chubut	0.1279	1.14
**27**	Isla Gaviota^a,c,r^	**IG**	45°06′S, 65°58′W	Chubut	-0.0498	0.95 (0.951 – 0.952)*
**28**	Isla Vernaci Norte^a,c,d,r,s^	**IVN1**	45°11′S, 66°30′W	Chubut	0.0058	1.01 (0.99 – 1.03)
**29**	Isla Vernaci Norte2^a,c,d,r,s^	**IVN2**	45°11′S, 66°30′W	Chubut	0.0112	1.01 (1.00 – 1.02)
**30**	Isla Vernaci Este^c,d,r,s^	**IVE**	45°11′S, 66°29′W	Chubut	-0.0216	0.98 (0.96 – 1.00)
**31**	Isla Vernaci Noroeste^a,c,r,s^	**IVNO**	45°10′S, 66°31′W	Chubut	0.1136	1.12 (1.03 – 1.21)*
**32**	Isla Vernaci Sudoeste^a,c,r,s^	**IVSO**	45°11′S, 66°31′W	Chubut	-0.0195	0.98 (0.96 – 1.00)
**33**	Isla Vernaci Fondo1^a,c^	**IVF1**	45°11′S, 66°30′W	Chubut	-0.0022	1.00
**34**	Punta Pájaros^t,v^	**PPaj**	46° 57’S, 66°51’W	Santa Cruz	0.0394	1.04
**35**	Isla Chaffers^t,u,v^	**ICh**	47°46’S, 65°52’W	Santa Cruz	0.0009	1.00 (0.99 – 1.01)
**36**	Isla Pájaros^t,u,v^	**IPa**	47°45’S, 65°58’W	Santa Cruz	0.0197	1.02 (1.01 – 1.03)*
**37**	Isla Quiroga^t,u,v^	**IQ**	47°45’S, 65°56’W	Santa Cruz	0.0334	1.03 (1.03 – 1.04)*
**38**	Isla Larga^t,v^	**ILg**	47°45’S, 65°56’W	Santa Cruz	0.0896	1.09
**39**	Islote Cañadon del Puerto^t,v^	**ICP**	47°45’S, 66°00’W	Santa Cruz	-0.0109	0.99
**40**	Isla del Rey^t,v^	**IdR**	47°46’S, 66°03’W	Santa Cruz	-0.0138	0.99
**41**	Islote Burlotti^t,v^	**IBti**	47°46’S, 65°57’W	Santa Cruz	0.0098	1.01
**42**	Isla Pingüino^t,v^	**IPno**	47°54’S, 65°43’W	Santa Cruz	-0.0177	0.98
**43**	Isla Chata^t,v^	**ICta**	47°56’S, 65°44’W	Santa Cruz	0.0312	1.03
**44**	Isla Schwarz^t,v^	**ISch**	48°04’S, 65°54’W	Santa Cruz	-0.0053	0.99
**45**	Islote Burgos^t,v^	**IteB**	48°05’S, 65°54’W	Santa Cruz	-0.0235	0.98
**46**	Isla Liebres^t,v^	**ILi**	48°06’S, 65°54’W	Santa Cruz	-0.0277	0.97
**47**	Punta Medanosa^t,v^	**PM**	48°06’S, 65°55’W	Santa Cruz	-0.0519	0.95
**48**	Punta Sur^t,v^	**PS**	48°07’S, 65°56’W	Santa Cruz	0.0253	1.03
**49**	Estancia 8 de Julio^t,u,v^	**E8J**	48°07’S, 66°08’W	Santa Cruz	0.0490	1.05 (1.04 – 1.06)*
**50**	Isla Rasa Chica^t,v^	**IRC**	48°22’S, 66°20’W	Santa Cruz	-0.0466	0.95
**51**	Islote del Bajío^t,v^	**Iba**	48°21’S, 66°21’W	Santa Cruz	0.1149	1.12
**52**	Islote sin Nombre^t,v^	**IsN**	48°22’S, 66°21’W	Santa Cruz	-0.0224	0.98
**53**	Cabo Guardián^t,u,v^	**CG**	48°21’S, 66°21’W	Santa Cruz	0.0142	1.01 (1.01 – 1.02)*
**54**	Banco Cormorán^t,v^	**BC**	49°16’S, 67°40’W	Santa Cruz	0.0081	1.01 (0.99 – 1.02)
**55**	Banco Justicia^t,v^	**BJ**	49°17’S, 67°41’W	Santa Cruz	-0.0433	0.96
**56**	Isla Leones (Sta Cruz)^t,v^	**IL(SC)**	50°04’S, 68°26’W	Santa Cruz	0.0053	1.01
**57**	Punta Entrada^t,v^	**PE**	50°08’S, 68°22’W	Santa Cruz	-0.0128	0.99
**58**	Monte León^t,v^	**ML**	50°22’S, 68°53’W	Santa Cruz	0.0137	1.01 (1.01 – 1.02)*
**59**	Isla Deseada^t,v^	**ID**	51°34’S, 69°02’W	Santa Cruz	0.0036	1.00 (0.97 – 1.03)
**60**	Cabo Vírgenes^t,v^	**CV**	52°22’S, 68°24’W	Santa Cruz	0.0132	1.01 (1.01 – 1.02)*
**61**	Isla Martillo^c,u,x^	**IMar**	54°54’S, 67°22´W	Tierra del Fuego	0.0624	1.06 (1.04 – 1.09)*
**62**	Bahía Franklin^c,w^	**BF**	54°52’S, 64°42’ W	Tierra del Fuego	0.1076	1.11
**63**	Isla Gofré^c,y^	**IGo**	54°42′S, 64°14′W	Tierra del Fuego	-0.0634	0.94
**64**	San Juan de Salvamento^z^	**SJS**	54º43´S, 63º48´W	Tierra del Fuego	0.0733	1.08
**65**	Islas Malvinas^aa^	**IM**	51°47’S, 59°31’W	Islas Malvinas	-0.0855	0.92

Confidence intervals are shown for those with more than three population data points. (*) indicate statistically significant trends. References: ^a^^7^, ^b^ A. Velázquez Guarda Ambiental ANPBSA (pers. comm.), ^c^^1^, ^d^^25^, ^e^Our data, ^f^^32^, ^g^^22^, ^h^^37^, ^i^^30^, ^j^^14^, ^k,u^^29^, ^l^^20^, ^m^^31^, ^n^^33^, ^o^^25,34^, ^p^^35^,^q^^36^,^r^^8^, ^s^^26^, ^[t9^, ^v^^23^, ^w^^58^, ^x^^38^, ^y^^39^, ^z^^24^, ^aa^^28^.

**Fig. 1:**
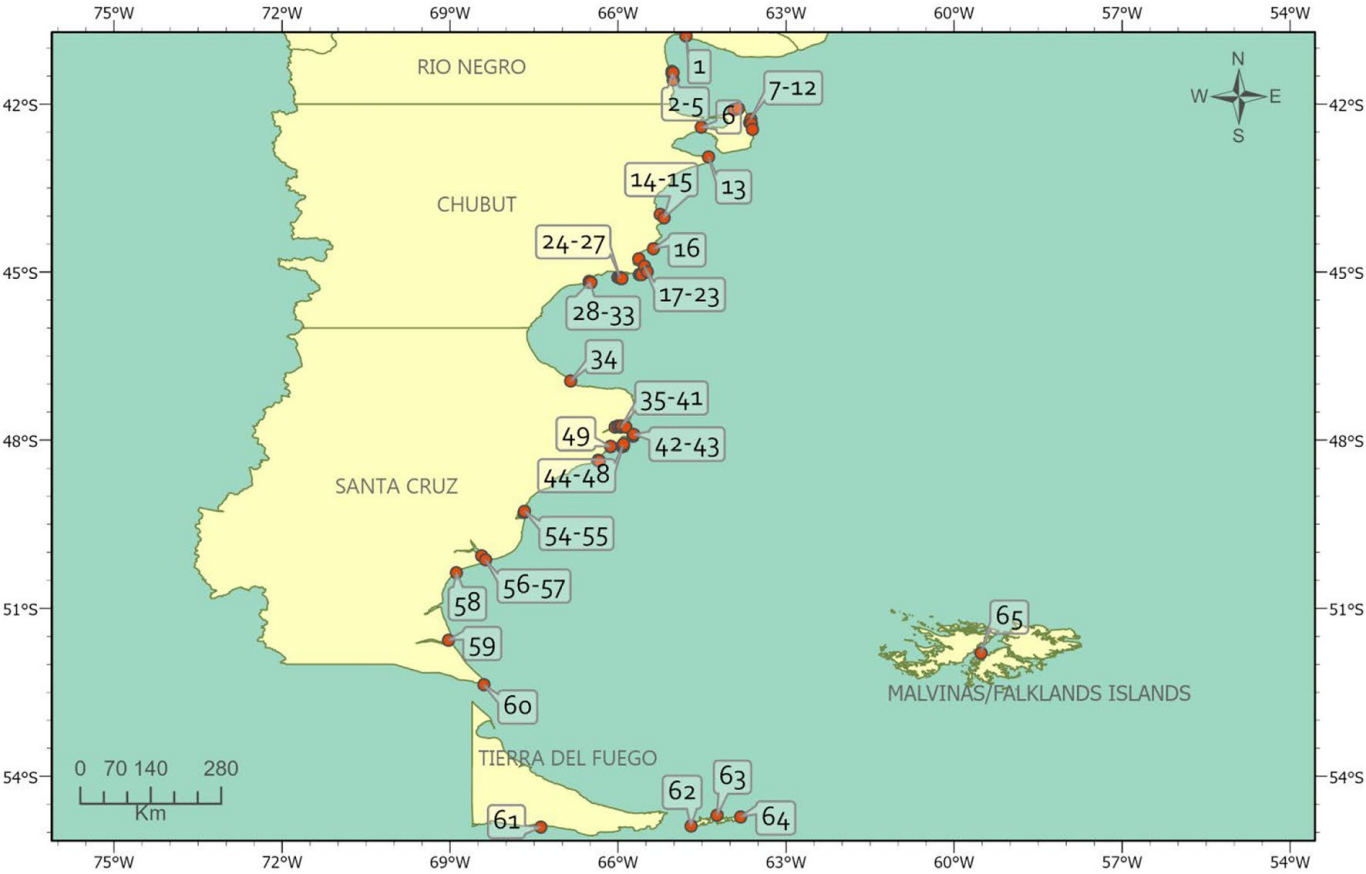
Geographic distribution of Magellanic penguin colonies along the Argentine Patagonian coast (orange points). Numbers correspond to colony IDs listed in Table 1.

All consulted abundance records and their original sources are listed in Table 1, covering colonies distributed along the entire Argentine Atlantic coast—from Río Negro to Tierra del Fuego—as well as the Malvinas/Falkland Islands (Figure 1).

### Field-based abundance estimation

For the colonies located within Islote Lobos Nacional Park, Río Negro Province, data were collected during field surveys conducted in the 2022/2023, 2023/2024, and 2024/2025 breeding seasons. Abundance estimates were obtained in October of each year, coinciding with the peak of egg laying and maximum nest occupancy, following standard census protocols for Magellanic penguin colonies^2,29^. A systematic sampling design was employed, in which active nests—defined as those containing an adult or a pair with eggs—were counted within circular plots of 100 m^2^, spaced every 50 meters along fixed parallel transects separated by 100 meters^29^. Each plot was delineated using a 5.65-meter rope, with one observer positioned at the center holding the rope and another walking the perimeter to identify all active nests within the area^40,8^. Permanent sampling plots were established during the first field season and resurveyed in all subsequent seasons. A total of 29 plots were established in Islote La Pastosa, 9 in Islote Redondo, and 9 in Isla de los Pájaros.

To define the spatial boundaries of each colony, the outermost nests were georeferenced, and total area was calculated using Geographic Information System (GIS) tools (ArcGIS, Esri Inc.). Nest density in unsampled areas within the three colonies monitored in Islote Lobos was estimated using natural neighbor interpolation^41^, allowing for a continuous spatial representation of abundance. For the remaining colonies, abundance values were taken directly from published literature and technical reports.

### Population trends

Data from 73 colonies were compiled, including three colonies monitored directly between 2022 and 2025, geographically distributed across four Argentine provinces: 5 colonies in Río Negro, 34 in Chubut, 28 in Santa Cruz, and 4 in Tierra del Fuego. The Malvinas/Falkland Islands were incorporated as a single analytical unit because most historical abundance estimates are reported at the scale of the entire archipelago, without sufficient temporal or spatial resolution to distinguish individual breeding sites. Although Bingham^28^ describes the use of fixed plots in multiple locations, the published abundance values are presented as aggregated estimates for the whole archipelago rather than as independent census totals for each colony. Earlier studies provide approximate abundance values for broad periods (e.g.,^42–44^, but they do not report year-specific counts or methodologically comparable time series. For this reason, only Bingham^28^ could be used to represent temporal change, as it is the only source reporting abundance estimates for distinct years.

Population trends were evaluated using two complementary approaches: (1) estimates restricted to the continental Argentine territory, which provide the primary basis for national-scale trend assessments, and (2) estimates that include the Malvinas/Falkland Islands. Although the Malvinas/Falkland Islands colony is part of the species’ geographic range, its markedly divergent population trajectory has the potential to disproportionately influence continental estimates and was treated analytically as a separate case to avoid bias.

Based on temporal data availability, colonies were classified into three groups: 32 colonies with three or more years of data, used to estimate log-linear population trends and confidence intervals via bootstrapping; 33 colonies with exactly two years of data, for which the finite population growth rate (λ) was calculated directly between both years; and 8 colonies with only a single abundance record (Punto Cero, Isla Cumbre, Isla Moreno, Isla Vernaci Fondo 2, Isla Viana Mayor, Isla Quinta, Isla Elena, and Isla Observatorio), which were excluded from trend, abundance, and projection analyses.

Based on this classification, a total of 65 Magellanic penguin colonies along the Argentine coastline were analyzed, including all colonies with at least two years of abundance data. Colonies with only two temporal records (≈45% of all identified colonies) were included to ensure broad national spatial coverage and avoid excluding a substantial proportion of known breeding sites. For each colony, the finite population growth rate (λ) was estimated by fitting a discrete-time, density-independent exponential model under the assumption of multiplicative error:

$${N}_{t}= {N}_{0} . { \lambda }^{\left(t-{t}_{0}\right)}$$

Where $N_t$ represents the estimated abundance in year *t*, *N₀* is the initial abundance, and *λ* is the finite rate of population growth^45^. Colonies were classified as having a positive trend if λ > 1, a negative trend if λ < 1, and a stable trend λ = 1. For colonies with three or more abundance records over time, the data were log-transformed and a linear regression was applied to estimate the instantaneous growth rate (*r*) using the equation:

$$r=\mathrm{ln}\left(\lambda \right)$$

From this, the finite population growth rate was obtained as: *λ*= *e*^*r*^*.* Confidence intervals (CI) for the growth rate were calculated via bootstrap resampling of the model residuals^46^.

For colonies with exactly two abundance records, the finite population growth rate *λ* was calculated directly between both points using the following formula:

$$\lambda = \left( {{\raise0.7ex\hbox{${N_{t} }$} \!\mathord{\left/ {\vphantom {{N_{t} } {N_{0} }}}\right.\kern-0pt} \!\lower0.7ex\hbox{${N_{0} }$}}} \right)^{{{\raise0.7ex\hbox{$1$} \!\mathord{\left/ {\vphantom {1 {t - t_{0} }}}\right.\kern-0pt} \!\lower0.7ex\hbox{${t - t_{0} }$}}}}$$

No confidence intervals were computed for these cases due to the limited data.

#### Estimation of overall population trend

To estimate an overall population trend over time, two approaches were applied: a simple weighted method, which maximizes spatial coverage by incorporating all colonies, and an empirical Bayesian approach, which provides a statistically more precise estimate for colonies with sufficient temporal replication. The weighted integration of colony-level trends follows general demographic and meta-analytic principles^45^, while the empirical Bayesian framework was adapted from previously established applications in pinniped population analyses^47^.

*a) Simple weighting method: *In this approach, the weighted mean of colony-specific population growth rates (λ) was calculated. The weight of each colony was defined as proportional to the number of years with available data and its mean abundance. This method was applied to both the 32 colonies with three or more data points and the 33 colonies with only two abundance records.

The weight for each colony (${w}_{i}$) was defined as follows:

$${w}_{i}= {\overline{N} }_{i} . {Y}_{i}$$

where ${\overline{N} }_{i}$ is the mean abundance of colony *i*, and *Y* is the number of years with available data.

Then, the weighted average of the colony-specific logarithmic growth rates (${r}_{i}= \mathrm{log}\left({\lambda }_{i}\right)$), was calculated, and from this value the overall population growth rate (${\lambda }_{general}$) was derived.

$$r_{general} = \frac{{\sum r_{i} . w_{i} }}{{\sum w_{i} }},\lambda_{general} = e^{{r_{general} }}$$

*b) Empirical Bayesian Method:* This approach was applied exclusively to the 32 colonies with three or more data points, as it requires a reliable estimate of the error associated with the trend. The procedure followed the methodology proposed by Calkins et al.^48^, adjusting individual trends based on their statistical precision and their relative weight within the total population.

• Calculation of the Bayesian weight, defined as:

$${w}_{tb}= \raisebox{1ex}{$vt$}\!\left/ \!\raisebox{-1ex}{$\left(vt+{\tau }^{2}\right)$}\right.$$

where $w_{tb}$ is the bayesian weight for a colony’s trend,$vt$ is the variance of the colony-specific trend, and ${\tau }^{2}$ is an estimate of the among-colony variance, calculated as ${\tau }^{2}={V}_{t}-{\overline{v} }_{t}$, where ${V}_{t}$ is the variance among the estimated trends of all colonies and ${\overline{v} }_{t}$ is the mean of the individual variances.

• Adjustmentof the trend and its variance for each colony:

$$t_{b} = \bar{t}.w_{{tb}} + t.\left( {1 - w_{{tb}} } \right)$$

$${v}_{tb}= {w}_{tb} . {\tau }^{2}$$

where ${t}_{b}$ and ${v}_{tb}$ are the Bayesian-adjusted trend and variance, $t$ is the colony-specific estimated trend, and is $\overline{t }$ he mean of all estimated trends.

• Calculation of the average population weight, ${w}^{A}$:

$$w_{i}^{A} = ~\frac{1}{{T_{i} }}~\mathop \sum \limits_{{t \in T_{i} }} \left( {\frac{{C_{{i,t}} }}{{\mathop \sum \nolimits_{j} C_{{j,t}} }}} \right)$$

where ${C}_{i,t}$ is the abundance of colony *i* in year *t*, and ${T}_{i}$ is the number of years with available data for that colony.

• Estimation of the adjusted general population trend:

$${t}_{b-A}= \sum \left({w}^{A}.{t}_{b}\right)$$

$${v}_{b-A}= \sum \left({w}^{A}.{t}_{b}\right)$$

where ${t}_{b-A}$ is the adjusted general population trend and ${v}_{b-A}$ is the total adjusted variance associated with that trend. Both are calculated as the weighted average of the colony-specific Bayesian-adjusted trends $({t}_{b})$ and their corresponding variances $({v}_{tb})$, using the abundance-based weight ${w}^{A}$ as the weighting factor. The abundance-based weights ${w}^{A}$ are normalized to sum to 1 across colonies; therefore, the expression ${t}_{b-A}= \sum \left({w}^{A}.{t}_{b}\right)$ represents a weighted average rather than a raw sum.

### Estimation of total abundance

To estimate the total number of breeding pairs along the Argentine coast, abundance values were interpolated for the years 1996, 2006, and 2016 using colony-specific population growth rates (λ) previously estimated. These years were selected because the period between the mid-1990s and mid-2010s includes the highest data availability, maximizing the coverage and representativeness of the historical series.

Interpolation was based on a discrete-time exponential growth model fitted to the observed abundance values for each colony.

Colonies for which the first recorded data point was after the target year were not extrapolated backward to avoid unsupported overestimations.

#### Projection to the year 2024

Abundance values for each colony were projected to the year 2024 using the same discrete-time exponential growth model employed for historical interpolation. For this, the most recent actual abundance value available for each colony and its respective estimated growth rate (λ) were used as the basis for projection.

Projected abundances were aggregated by colony groups (2 data points and ≥3 data points), as well as combined to estimate an overall population abundance for the year 2024.

For the colonies El Pedral (EP) and Estancia San Lorenzo (ESL), it was observed that exponential models fitted to the entire data series produced excessively high projections due to very elevated growth rates during the early stages of colony establishment. Therefore, λ values were recalculated using only the most recent data, corresponding to more stable phases of population growth. These adjusted rates were then used for interpolating and projecting abundances in 2016 and 2024, allowing for more realistic and consistent estimates aligned with the current population dynamics of these colonies.

Additionally, projections to 2024 were conducted both including and excluding the Malvinas/Falkland Islands colony, in order to explore its influence on the total abundance estimation.

All data processing and analysis were conducted in R v4.3.1 (R Core Team, 2023). The packages tidyverse^49^, ggplot2^50^ were used for data manipulation, visualization, and estimation of population growth rates (λ), including log-linear fits and exponential projections. Cartographic outputs were produced using ArcGIS Pro v3.5.1^51^.

## Results

### Colony-specific population trends

**Fig. 2:**
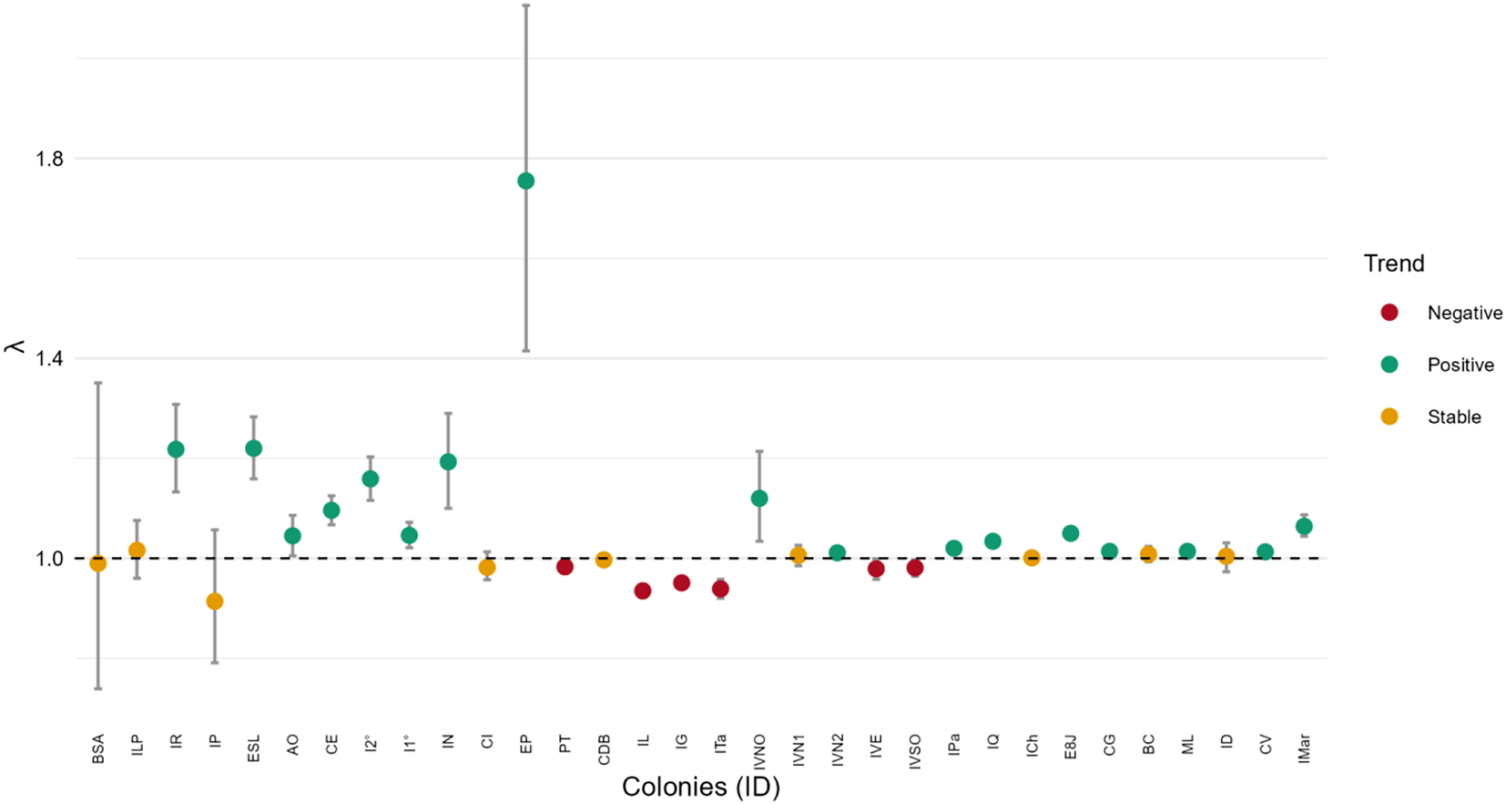
Colonies with at least three abundance estimates, classified by population trend—positive (green), negative (red), or stable (yellow)—and ordered by latitude (north to south).

**Fig. 3:**
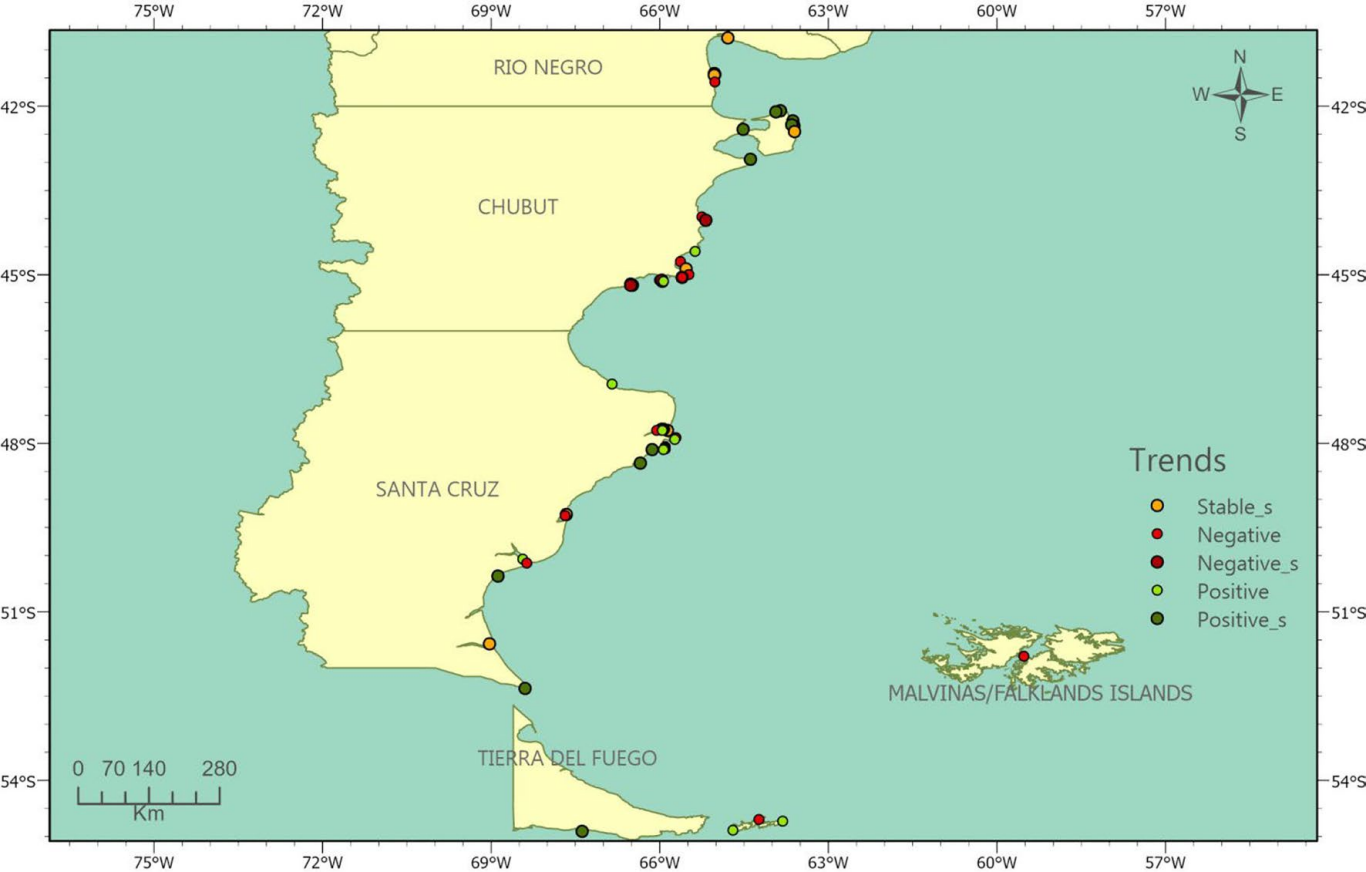
Magellanic penguin colonies classified according to their population trend (λ) along the Argentine coast. Categories with the suffix “_s” indicate statistically significant trends: growth (“Positive_s”), decline (“Negative_s”), or stability (“Stable_s”). The remaining colonies show non-significant trends based on only two abundance estimates.

Among the 65 colonies analyzed, 33 showed an increase in abundance and 32 exhibited a decline between the available census records (Table 1; Supplementary Figure 1). Within this dataset, the 32 colonies with three or more temporal records allowed statistically supported trend estimation, given their greater temporal replication (≥3 census years). Of these, 17 showed a statistically significant positive trend, 6 exhibited a significant negative trend, and 9 displayed non-significant (stable) trajectories (Figure 2). Across colonies with sufficient temporal data, both increasing and decreasing trajectories were documented. The spatial distribution of colony-specific population trends indicates that population changes are not homogeneous (Figure 3).

### General population trend

To estimate the population trend of the species at the national scale, population growth rates were calculated using different weighting methodologies, applied to three analytical groups: (1) all available colonies with at least two population records (n=65), (2) all colonies excluding the Malvinas/Falkland Islands colony (n=64), and (3) those colonies with data from three or more census years (n = 32), for which adjustments based on the statistical precision of the trend were possible.

**Fig. 4:**
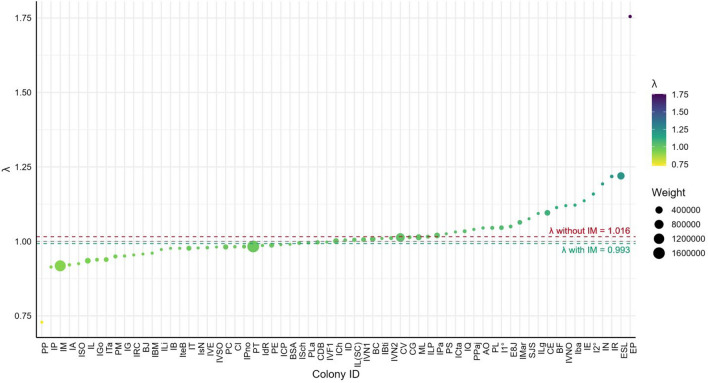
Population growth rates (λ) for all colonies, ordered by value and weighted using the simple method. Each point represents a colony, with symbol size proportional to its weighting factor. The dashed red line indicates the overall λ excluding the Malvinas/Falklands colony (IM), while the dashed green line represents the value including it.

Considering all 65 monitored colonies (including IM), the weighted overall growth rate was λ = 0.993, with a 95% confidence interval of [0.967 – 1.018] (Figure 4). Although the central estimate indicates a slight negative trend, the confidence interval includes 1, suggesting no statistically significant change and thus a stable population. This estimate is strongly influenced by the pronounced decline in the Malvinas/Falklands Islands colony (IM), where abundance dropped from approximately 1,300,000 breeding pairs in 1989–1990 to around 100,000 in 2019–2020 ^28^.

When this colony is excluded, the overall population growth rate increases to λ = 1.016 (95% CI: [0.988 – 1.040]) (Figure 4). In this case, although the point estimate indicates a positive trend, the confidence interval still includes 1, indicating that the continental metapopulation remains statistically stable. Thus, regardless of the inclusion or exclusion of IM, the overall trend is statistically stable, with directional variation depending on the colony set considered.

To validate the robustness of these results, an analysis was conducted using only the colonies with greater temporal support (≥3 data points, n=32), where it is possible to apply models that incorporate variance and precision estimates. Within this group, overall trends were estimated using two alternative methods.

**Fig. 5:**
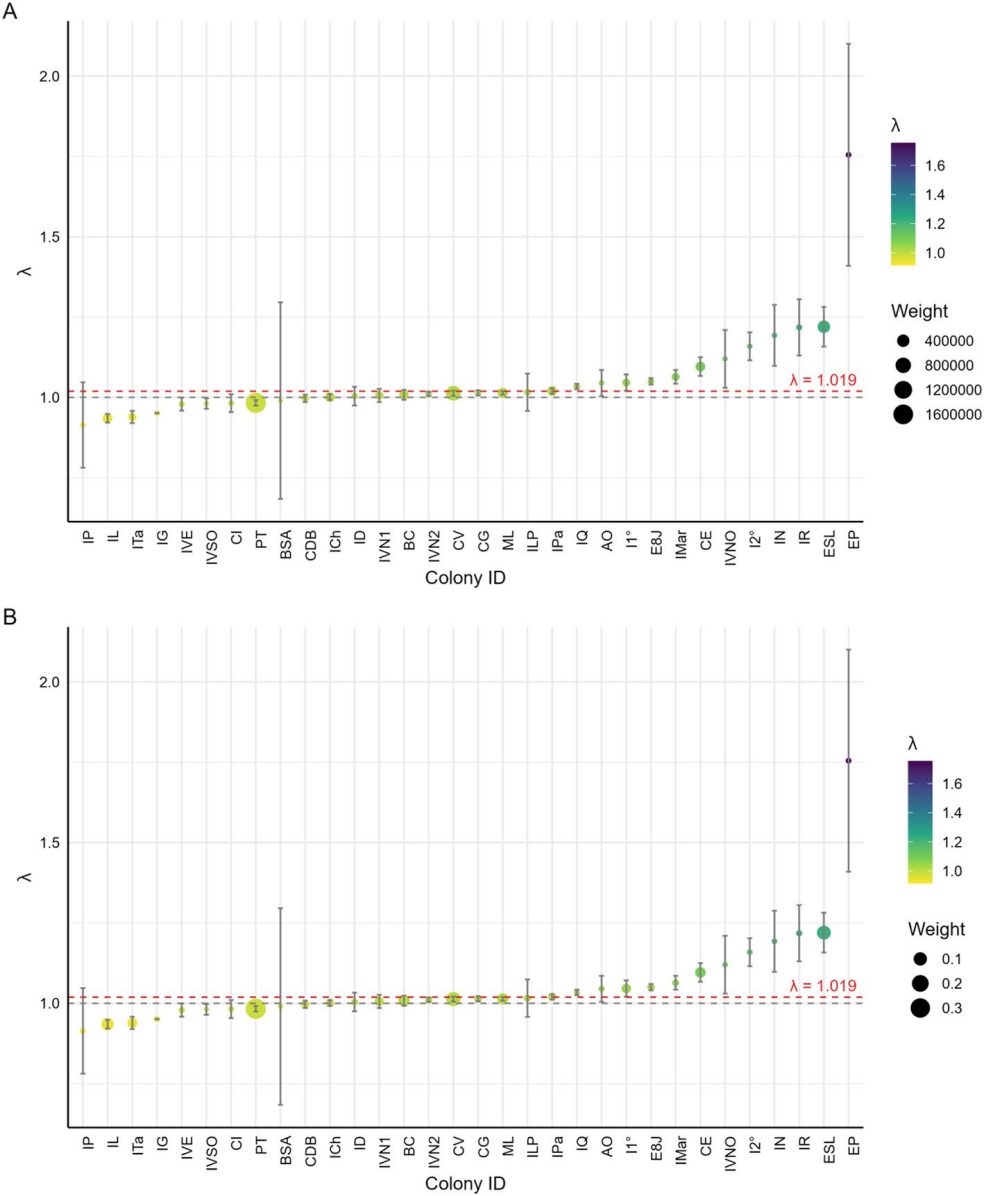
Finite population growth rates (λ) for colonies with three or more data points. Panel A shows λ values weighted using the simple method, and Panel B shows values weighted using the Bayesian method. Dot size is proportional to colony weight. The red dashed line indicates the overall weighted mean λ (λ = 1.019).

The first approach (simple weighting) consisted of a weighted average of individual λ values, assigning greater weight to larger and better-monitored colonies. The resulting estimate with the simple weighting approach was λ=1.01932 (95% CI: [0.989–1.051]) (Figure 5A). The second approach used a Bayesian weighting scheme in which the λ estimates were adjusted based on their statistical precision and the average size of each colony over time. This method reduces the influence of colonies with high uncertainty and is considered particularly appropriate in contexts of uneven data quality. The estimated rate under the Bayesian model was nearly identical to that obtained with the simple weighting method: λ = 1.0192 (95% CI: [0.994 – 1.045]) (Figure 5B).

### Temporal changes in total abundance

National-scale estimates of population abundance reveal a clear increase over the last three decades, culminating in higher projected values by 2024. This growth is evident even when accounting for variability among colonies and differences in temporal data coverage.

In 1996, the estimated number of breeding pairs for Argentine colonies (excluding the Malvinas/Falkland Islands colony) was approximately 863,670. This number gradually increased to 909,713 in 2006 and to 990,393 in 2016. The projection for 2024 estimates a total of 1,280,900 breeding pairs, representing a 48% increase compared to 1996 and a 29% increase compared to 2016.

**Fig. 6:**
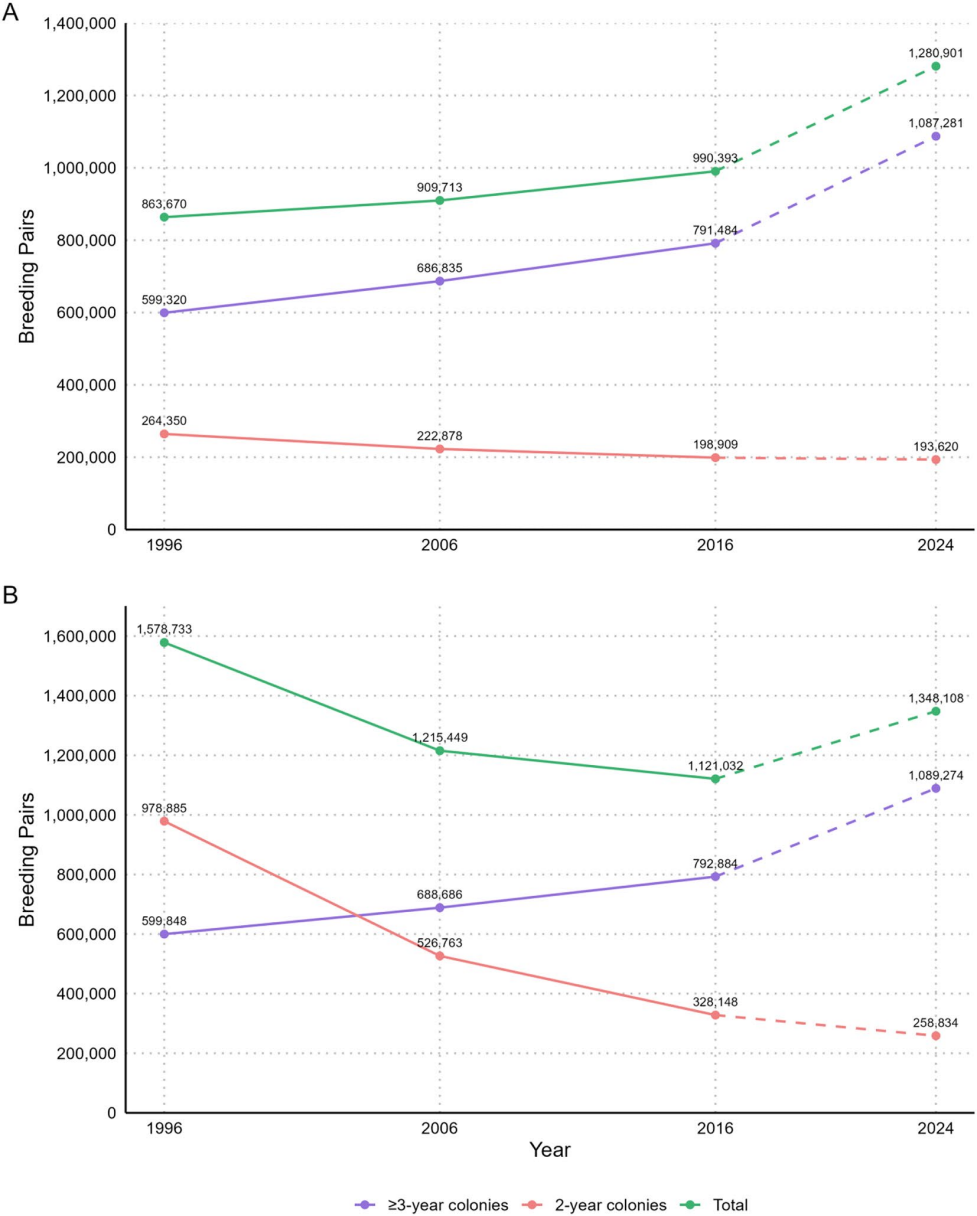
Estimated number of breeding pairs in Argentina from 1996 to 2024. **(A)** Estimates excluding the Malvinas/Falkland Islands, differentiated by colonies with ≥3 census data points, colonies with only 2 data points, and the national total. **(B)** Estimates including the Malvinas/Falkland Islands, showing the same grouping structure to illustrate the influence of this colony on total abundance.

This increase in abundance is largely associated with colonies for which at least three census records are available, allowing trend estimates with associated uncertainty (confidence intervals). These colonies increased from 599,320 pairs in 1996 to a projected 1,087,281 in 2024—an increase of over 80% in nearly three decades. In contrast, colonies with only two data points over the study period show a slight decline in projected abundance, from 264,350 pairs in 1996 to 193,620 in 2024 (Figure 6A). This difference may reflect actual local declines or the uncertainty and limitations of projections based on short time series. These spatial and temporal patterns are consistent with previously documented northward expansion and establishment of new breeding sites in northern Patagonia^25,7^.

When the Malvinas/Falkland Islands (IM) colony is included in the analysis (Figure 6B), the total projected abundance for 2024 rises to 1,348,100 breeding pairs. This means that the IM colony accounts for approximately 67,000 projected pairs at present. However, its inclusion has a more significant impact on the perception of historical trends, as in 1996 the estimate for this colony is 714,535 breeding pairs (based on data from^28^, indicating a population decline of 90% over three decades.

## Discussion

The results obtained suggest that, at a national scale and based on the colonies for which abundance information is available, the Magellanic penguin exhibits an overall stable population with a long-term positive tendency during the analyzed period.

The agreement between the two methodological approaches strengthens the reliability of the national-scale population trend estimate, beyond the weighting criterion adopted. Simple weighting directly reflects the demographic contribution of each colony, giving more weight to those that are larger and monitored more frequently, which is useful for capturing aggregate trends at the national level. The Bayesian model, on the other hand, incorporates the uncertainty of each estimate, attenuating the influence of colonies with high variance or scarce data, resulting in a more conservative and robust estimate—particularly useful in contexts of high heterogeneity and variable data quality, as in this case. Taken together, these complementary approaches allow a confident interpretation of the aggregate trend signal.

Although the point estimate of the growth rate (λ=1.019) suggests an average annual increase close to 2%, the associated confidence interval includes 1, indicating that this positive trend is not statistically significant. Therefore, the population can be considered stable, with an apparent upward tendency over the analyzed period.

The national-scale results suggest that the species maintains a strong and expanding breeding population in Argentina, underscoring the need for systematic monitoring to anticipate potential demographic changes. The sharp decline recorded in the Malvinas/Falkland Islands does not significantly alter the current outlook of national abundance but highlights the importance of differentiated and more regular monitoring of this colony. The 2024 projection suggests that the species has reached a new population threshold, consolidating its presence along its entire distribution in Argentina.

Several previous studies have addressed the assessment of population trends of Magellanic penguins along the Argentine Patagonian coast. The study by Gandini et al.^29^ was one of the first comprehensive assessments, focused on the provinces of Chubut, Santa Cruz, and Tierra del Fuego, at a time when many colonies in the northern portion of the current distribution (such as those in Río Negro) did not yet exist, and others in the far south were not yet established (e.g., San Juan de Salvamento^24^. That study estimated approximately 652,000 breeding pairs across the 36 colonies surveyed. More recently, Millones et al.^9^ conducted a similar study for the province of Santa Cruz, analyzing 27 colonies in total. For the period 2014–2017, the reported number of breeding pairs was approximately 353,000, maintaining a relatively stable population compared to the 1990s, when it was estimated at 315,000 pairs.

In a similar vein, García Borboroglu et al.^7^ updated the status of colonies in northern Patagonia, detecting a population increase of 19.7% over 20 years and a marked redistribution towards more northern latitudes. Their analysis highlighted a transformation at a regional structure, with growth exceeding 700% in Río Negro and northern Chubut, while many colonies in central and southern Chubut showed signs of stagnation or decline.

Our results confirm and expand these patterns at a national scale. Based on the analysis of 64 colonies distributed along the entire Argentine coast, we estimate an approximate long-term increase of ~48% over the study period, based on interpolated and projected abundance values derived from colony-specific growth rates (λ). This broader coverage reveals a more heterogeneous picture, where growth in northern colonies coexists with stability or decline in historically established southern areas. This broader spatial pattern is consistent with previous findings suggesting that processes of dispersal, the establishment of new colonies, and local environmental conditions play a key role in shaping the spatial and temporal dynamics of Magellanic penguin populations.

Regarding the colonies located in the Malvinas/Falkland Islands, the evaluation of their population dynamics over time is constrained by the limited availability of systematic monitoring, as well as by substantial discrepancies among published abundance estimates, which often lack detailed methodological information. In the mid-1980s, Croxall et al.^43^ estimated a population of around 100,000 breeding pairs, based on a broad survey of colonies. However, the authors themselves acknowledged limitations associated with data quality and access difficulties to some remote areas.

A significant inconsistency emerges when comparing different sources for the same period. While Bingham^28^ reports that the breeding population in the Malvinas/Falkland Islands reached 1.3 million pairs in 1989/90, Pütz et al.^44^, compiling survey data from 1994/95—citing Bingham^52^—reported an estimate of approximately 100,000 pairs, consistent with values from Croxall et al.^43^. This large discrepancy suggests that the 1.3 million figure may have been retrospectively reconstructed, lacking detailed methodological support in contemporary publications. Furthermore, Bingham’s publications lack essential details such as the location of fixed plots, sampled area, and the number of nests recorded, which severely limits the ability to assess the robustness and replicability of his estimates.

Nonetheless, in the same work, Bingham^28^ hypothesizes that part of the decline in the Malvinas/Falkland Islands could be related to population redistribution, with possible movements toward continental colonies. In particular, population increases have been reported in Cabo Vírgenes and Isla Contramaestra (Chile), where favorable environmental conditions and the presence of fishing exclusion zones may have facilitated settlement^28^.

Adding to this controversy, Bertellotti^42^ reported that the breeding population of Magellanic penguins in the Malvinas/Falkland Islands may have undergone one of the steepest declines in recent decades. According to his data, a population estimated at nearly 500,000 breeding pairs in 1980 may have declined to less than a quarter of that by 2002, continuing its downward trend likely due to commercial fishing. This observation not only reinforces the diagnosis of sustained decline but also highlights the lack of coherence among available figures, further complicating historical reconstruction of population dynamics in the region.

Croxall et al.^43^, Pütz et al.^44^, Bertellotti^42^, and Bingham^28^ all point to a sustained decline in the breeding population of Magellanic penguins in the Malvinas/Falkland Islands, although the reported figures differ significantly. These discrepancies reflect the lack of systematic monitoring and the limited availability of verifiable data for the region. The compiled information suggests that the long-term trajectory of the breeding population in the archipelago is consistent with a declining trend, even if the magnitude of that decline remains uncertain due to methodological limitations and the absence of standardized, long-term monitoring programs.

The observed variation in population trajectories among colonies suggests that local environmental conditions, demographic regulation, and site-specific processes may differentially influence abundance over time, generating both positive and negative changes depending on the ecological and anthropogenic context of each site. This heterogeneity indicates that population dynamics are not uniformly driven by a single external factor but rather emerge from multiple interacting mechanisms operating at local scales.

Several authors have mentioned fishing as a potential threat to the conservation of Magellanic penguins, either through incidental mortality or trophic competition^4,53^. However, studies specifically focused on these interactions^16,54–56^ reported very low mortality rates for the main coastal and offshore fisheries in Patagonia (shrimp and hake fisheries). Incidental catches of Magellanic penguins were also very low in other coastal and small-scale fisheries in northern Patagonia, Uruguay, and Brazil^57^.

Regarding trophic competition, Gandini et al.^16^ reported that penguin diet composition indicates little competition with the commercial shrimp fishery because the target species of the fishery is not exploited by penguins. A significant degree of trophic overlap, however, was found between the by-catch of this fishery and the penguins’ diet, primarily involving anchovy and hake. Nevertheless, this interaction does not appear to have population-level consequences. Both anchovy and hake have maintained stable fish stocks, with no evidence of conservation concerns for hake in the last two decades and no historical indication of decline for anchovy. These patterns suggest that prey availability for penguins has likely remained stable and not measurably reduced by fishery exploitation. Similarly, incidental mortality during fishing operations was low, with annual mortality rates estimated to be below 1%.

In short, although fishing is frequently mentioned as a conservation threat, the available information is limited, heterogeneous, and in many cases outdated. A more detailed and updated evaluation of these interactions is needed to determine whether fishing is exerting measurable effects on penguin populations at a regional scale.

Our results consolidate the evidence that the population of this species in continental Argentina is undergoing a phase of expansion, albeit within a context of marked regional heterogeneity. This broad perspective allows local trajectories to be contextualized within an overall pattern of growth, without losing sight of those colonies showing signs of stagnation or decline. In this framework, improving both the coverage and quality of available data is essential to refine population monitoring and support robust national-scale assessments.

Although the national trend is statistically stable, the observed heterogeneity among colonies highlights the importance of continuing standardized regional monitoring programs to support early detection of local declines and inform adaptive conservation planning.

In addition, a more comprehensive demographic understanding would benefit from the systematic collection of parameters that were not assessable in this study — such as mortality, breeding success, recruitment, immigration and emigration — to better identify the mechanisms shaping local trends and strengthen long-term conservation planning.

In conclusion, the general interpretation that emerges from our results indicates that: the Magellanic penguin population along the Argentine coast exhibits a statistically stable trend, with no evidence of collapse or widespread decline.

## Supplementary Information

Supplementary Information.

## Data Availability

The datasets generated and/or analyzed during the current study are not publicly available due to conservation restrictions and the extensive compilation effort involved, but are available from the corresponding author on reasonable request.
