# Supplementary material for: Status and global population trend of the Magellanic penguin Spheniscus magellanicus along the Argentine coast
Source: Sci Rep. 2026 Jan 10;16:3743. doi: 10.1038/s41598-025-33756-3 (PMC12852185; doi:10.1038/s41598-025-33756-3)
Supplement: Supplementary file 1 — Supplementary Information. [file 41598_2025_33756_MOESM1_ESM.pdf]

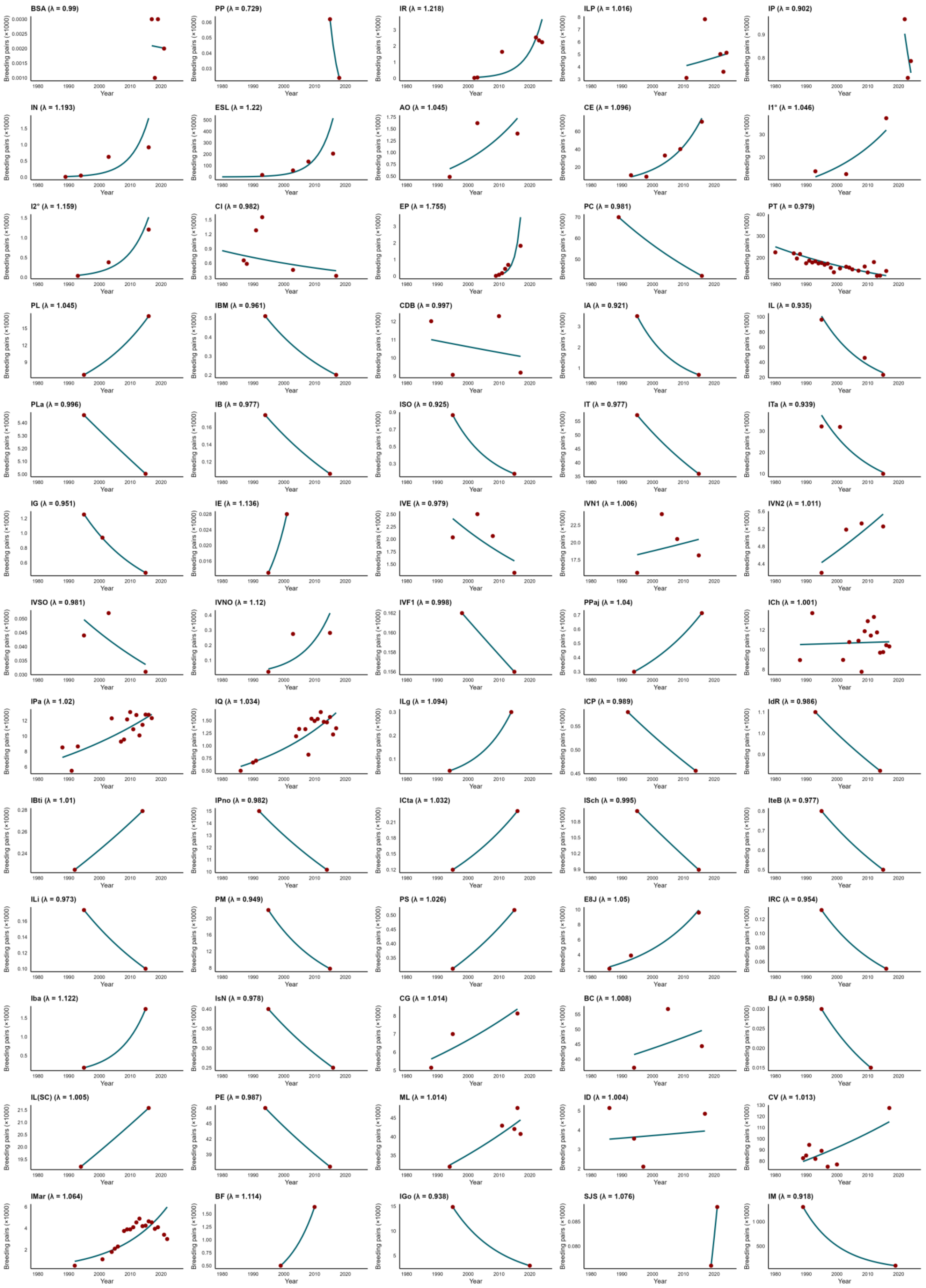

Figure S1. Log-linear population trends fitted for all 65 colonies analyzed. Each panel shows the observed breeding pair estimates (red dots), the fitted population trajectory (blue line), and the estimated finite population growth rate (λ).
